# Intravesicular Phosphatase PHOSPHO1 Function in Enamel Mineralization and Prism Formation

**DOI:** 10.3389/fphys.2017.00805

**Published:** 2017-10-17

**Authors:** Mirali Pandya, Lauren Rosene, Colin Farquharson, José L. Millán, Thomas G. H. Diekwisch

**Affiliations:** ^1^Department of Periodontics, Texas A&M College of Dentistry, Dallas, TX, United States; ^2^Center for Craniofacial Research and Diagnosis, Texas A&M College of Dentistry, Dallas, TX, United States; ^3^Division of Developmental Biology, The Roslin Institute and The Royal (Dick) School of Veterinary Studies, University of Edinburgh, Edinburgh, United Kingdom; ^4^Sanford Children's Health Research Center, Sanford-Burnham Institute for Medical Research, La Jolla, CA, United States

**Keywords:** amelogenesis, PHOSPHO1, ameloblast, enamel, matrix vesicle

## Abstract

The transport of mineral ions from the enamel organ-associated blood vessels to the developing enamel crystals involves complex cargo packaging and carriage mechanisms across several cell layers, including the ameloblast layer and the stratum intermedium. Previous studies have established PHOSPHO1 as a matrix vesicle membrane-associated phosphatase that interacts with matrix vesicles molecules phosphoethanolamine and phosphocholine to initiate apatite crystal formation inside of matrix vesicles in bone. In the present study, we sought to determine the function of *Phospho1* during amelogenesis. PHOSPHO1 protein localization during amelogenesis was verified using immunohistochemistry, with positive signals in the enamel layer, ameloblast Tomes' processes, and in the walls of ameloblast secretory vesicles. These ameloblast secretory vesicle walls were also labeled for amelogenin and the exosomal protein marker HSP70 using immunohistochemistry. Furthermore, PHOSPHO1 presence in the enamel organ was confirmed by Western blot. *Phospho1*^−/−^ mice lacked sharp incisal tips, featured a significant 25% increase in total enamel volume, and demonstrated a significant 2-fold reduction in silver grain density of von Kossa stained ground sections indicative of reduced mineralization in the enamel layer when compared to wild-type mice (*p* < 0.001). Scanning electron micrographs of *Phospho1*^−/−^ mouse enamel revealed a loss of the prominent enamel prism “picket fence” structure, a loss of parallel crystal organization within prisms, and a 1.56-fold increase in enamel prism width (*p* < 0.0001). Finally, EDS elemental analysis demonstrated a significant decrease in phosphate incorporation in the enamel layer when compared to controls (*p* < 0.05). Together, these data establish that the matrix vesicle membrane-associated phosphatase PHOSPHO1 is essential for physiological enamel mineralization. Our findings also suggest that intracellular ameloblast secretory vesicles have unexpected compositional similarities with the extracellular matrix vesicles of bone, dentin, and cementum in terms of vesicle membrane composition and intravesicular ion assembly.

## Introduction

Amelogenesis is a complex process that involves a multitude of proteins and proteinases to facilitate the orderly assembly of elongated calcium phosphate apatite crystals into enamel prisms. Early studies have established that proline/glutamine-rich enamel proteins such as amelogenin, ameloblastin, and enamelin control enamel crystal shape and habit by facilitating enamel crystal growth in c-axis dimension and limiting their expansion in width (Diekwisch et al., 1993; Masuya et al., 2005; Hatakeyama et al., 2009; Gopinathan et al., 2014). During the secretion of these structural proteins, two enamel proteases, MMP20 and KLK4, are involved in the processing and degradation of the proline-rich enamel matrix (Ryu et al., 2002; Shin et al., 2014). Through the step-wise removal of the organic protein matrix, the MMP20 and KLK4 enamel proteases facilitate inorganic crystal nucleation and growth resulting in an overall increase of enamel hardness (Lu et al., 2008). In addition, MMP20 appears to have additional functions related to the stability of the dentin-enamel junction, ameloblast retreat during enamel thickening, and ameloblast differentiation (Hu et al., 2016).

Enamel proteases not only affect enamel crystal growth by processing matrix proteins such as amelogenins that are directly in contact with the growing crystal surface. Enzymes also play a role as components of the vesicular transport machinery that facilitates the orderly movement of ions, notably calcium and phosphate, from the blood vessels of the enamel organ into the enamel layer. The secretory cells of bone, dentin, and cartilage connective tissues contain small vesicles surrounded by a lipid bilayer that in addition to small calcium phosphate crystals contain a number of enzymes that are important for their function in tissue mineralization, including tissue non-specific alkaline phosphate (TNAP), nucleotide pyrophosphatase phosphodiesterase (NPP1/PC-1), annexins (ANX), and other matrix metalloproteinases (MMPs; Hsu and Anderson, 1978; Anderson, 1984; Bonucci, 1992; Dean et al., 1992; Wuthier et al., 1992). These unique proteases have been implicated in the mineralization of bone and dentin (Golub, 2009).

One of the matrix vesicle enzymes involved in initiation of bone, dentin, and cartilage mineralization is the matrix vesicle phosphatase PHOSPHO1 (Millán, 2013). PHOSPHO1 has been identified in the matrix vesicles of osteoblasts, odontoblasts, and chondrocytes (Houston et al., 2004; Roberts et al., 2007; McKee et al., 2013). Biochemical studies have characterized PHOSPHO1 as a haloacid dehalogenase family member that interacts with matrix vesicle molecules phosphoethanolamine and phosphocholine to initiate apatite crystal formation inside of matrix vesicles (Millán, 2013; Cui et al., 2016). PHOSPHO1 has also been localized in ameloblasts (McKee et al., 2013), and enzyme replacement therapy has verified the essential role of the matrix vesicle alkaline phosphate TNAP for enamel formation (Yadav et al., 2012), but according to textbook knowledge, enamel mineralization occurs without the involvement of matrix vesicles (Nanci, 2008).

We thus conducted the present study to characterize the role of PHOSPHO1 during amelogenesis and to determine the function of PHOSPHO1 as a component of the ameloblast secretory vesicle wall in its effect on enamel structure and mineralization. We hypothesized that the loss of PHOSPHO1 would affect enamel structural integrity through a delay in intravesicular mineralization initiation, and we used light and electron microscopy as well as elemental mapping and von Kossa staining to characterize the effects of loss of PHOSPHO1 on the enamel layer. Our data not only provide a structural characterization of the *Phospho1* deficient enamel but also give insights into the function of the matrix vesicle phosphatase PHOSPHO1 related to enamel ion transport in vesicles.

## Materials and methods

### Animal models and biosafety

Animals were sacrificed in accordance with guidelines of the Animal Care and Use Committee at the Sanford Burnham Prebys Medical Discovery Institute and National Institutes of Health (NIH). Breeding, genotyping, and characterization of *Phospho1*^−/−^ mice has been described previously (Yadav et al., 2011; Zweifler et al., 2016). Eleven mandibles each from control and *Phospho1*^−/−^ mice were chosen for histology and microscopy studies. All research involving biohazards and toxins has been carried out according to Texas A&M institutional biosafety standards.

### Ultrathin ground sections of control and *Phospho1^−/−^* mouse mandibles

Thirty day old hemi-mandibles of control and *Phospho1*^−/−^ were fixed in 10% formalin and processed for ground sections. The mandibles were subjected to a series of different gradients of alcohol as well as mixture of ethanol/technovit as per the EXAKT company standard protocol for preparation of tissue samples for ground sections. Once the samples were in 100% light cure technovit (Technovit 7200, EXAKT), they were polymerized and embedded. The samples were grossly sectioned using a diamond bandsaw (EXAKT 300 CP), ground and polished to produce 30 μm thin sections (Liu et al., 2016). Two of the four ground sections were stained with alizarin red for 90 min and the other two for von Kossa staining.

### Radiographs

Thirty day old hemi-mandibles were analyzed using a Faxitron MX-20 specimen radiography system (Faxitron X-ray Corp., IL) at 20 kV for 20 s.

### Micro-CT analysis

Three WT and three *Phospho1*^−/−^ mouse mandibles were imaged individually and analyzed using a Micro-CT 20 Scanco Medical Scanner (Zürich, Switzerland). Specimens were scanned in standard resolution mode, with *x*-, *y*-, and *z*-axis resolution of ~10 μm. The level of X-ray exposure was 55 kVp energy and 800 ms exposure time. After the scanning was completed, 3-D data were generated by segmenting specimens at a threshold chosen to only include enamel tissue based on a morphological match with normal tooth histology. For each mandible, 70 slices were assembled and analyzed to perform a 3-D reconstruction of the incisors, and the total enamel volume fractions of the incisors were calculated and compared between the WT and *Phospho1*^−/−^ samples.

### Scanning electron microscopy (SEM) and energy dispersive X-ray spectroscopy (EDS)

Thirty day old hemi-mandibles for control and *Phospho1*^−/−^ mice were fixed in 10% formalin and treated gently with 4.5% EDTA for 15 min. The etched samples were dried and sputter coated with Au/Pd alloy for 90 s. These mandibles were further analyzed using a scanning electron microscope (JEOL JSM-6010LA). For EDS analysis, the samples were fixed, dried, and analyzed by selecting five random points in the enamel region. The elemental mapping at the selected points was recorded and the mass percent amount of calcium and phosphorous was compared between the control and PHOSPHO1 samples.

### Transmission electron microscopy

E16 tooth organs were cultured for 12 days, and thereafter fixed in Karnovsky's fixative as previously described (Diekwisch, 1998), dehydrated and embedded in Eponate 12 (Ted Pella, Redding, CA). Following polymerization of the Eponate, ultrathin sections were cut on a Leica Ultracut UCT ultramicrotome. After drying, sections were contrasted using uranyl acetate and Reynold's lead citrate for 15 min each. Stained sections were examined using a JEOL 1220EX transmission electron microscope at the UIC Research Resources Center (Chicago, IL).

### Paraffin sections and immunohistochemistry

Three days old hemi-mandibles of three wild-type mice were fixed in 10% formalin, decalcified with 4.5% EDTA for 1 week, and then processed for regular paraffin sectioning after dehydrating through graded series of ethanol and xylene. Alternatively, a second set of 3-days old wildtype mandibles was fixed in Bouin's fixative and also processed into 5 μm thin paraffin sections. For antigen retrieval, the paraffin fixed samples were incubated in 10 mM sodium citrate buffer for 30 min at pH 6 and boiling temperature. Alternatively, the samples fixed in Bouin's fixative were treated with 8M guanidine HCl overnight at 4°C and pH 8 to unmask the PHOSPHO1 antigen sites as suggested by McKee et al. (2013). Following antigen retrieval, samples were incubated using human recombinant Fab monoclonal anti-PHOSPHO1 primary antibody (AbD Serotec, Morphosys AG) or polyclonal anti-PHOSPHO1 antibody (GeneTex, USA) for PHOSPHO1 identification. Other vesicle associated proteins such as amelogenin or HSP70 were labeled using a polyclonal anti-amelogenin antibody or a mouse monoclonal anti-HSP70 antibody (Abcam, Cambridge, MA). Immunohistochemistry was performed using a broad spectrum IHC staining kit and an AEC substrate kit (Life Technologies, Carlsbad, CA) and stained sections were analyzed using a Leica DMR light microscope (Nuhsbaum, IL).

### Western blot

Enamel organs were immediately harvested from unerupted mandibular molars of 3 months old pigs sacrificed at a local animal farm. The enamel organs were subjected to extraction with 0.5% sodium dodecyl sulfate (SDS) lysis buffer with subsequent extraction with 4M guanidine HCl (pH 7.4). Initially, the proteins from enamel organ were extracted using SDS lysis buffer for 5 days. The supernatant was collected after centrifugation at 2,400 g and 4°C. The residue after SDS extraction was subjected to extraction with 4M guanidine (Gu) HCl buffer for 5 days. The supernatant from Gu-based extract was collected and processed following the same procedure as the SDS based extract. The extracted protein lysate from SDS and Gu-based extracts were dialyzed against double distilled water for 1 week. Equal amounts of protein extracts were loaded for both groups in a 10% SDS polyacrylamide gel and was subjected to gel electrophoresis at 150 V for 58 min. A semi-dry transfer system was used to transfer proteins from the gel to a polyvinylidine difluoride (PVDF) membrane at 18 V for 40 min. The PVDF membrane was blocked for 1 h with 5% dry milk in tris buffered saline with tween 20 (TBST), incubated with human recombinant Fab monoclonal anti-PHOSPHO1 primary antibody (1:500, AbD Serotec, Morphosys AG) for 1 h, followed by washing in TBST three times for 15 min and incubation with anti-mouse IgG HRP conjugated secondary antibody (1:2,000, cell signaling) for 1 h. The signal was detected using a chemiluminescent substrate (Thermo Scientific, USA).

### Statistical analysis

The width and the inter-prism space of enamel prisms was measured based on three control and three *Phospho1*^−/−^ samples. Measurement of prism width and interprismatic distance were obtained from scanning electron micrographs of etched mouse molar enamel cross sections. For our analysis, only mid-sagittal cross sections were chosen, and all samples were etched at identical times and concentrations, and rinsed for the same amount of time. Representative micrographs at 750x magnification from the distal interproximal enamel of the first molar were chosen for further analysis based on optimal prism pattern identification. Ten prisms were randomly chosen from each micrograph, and their width and the inter-prism space were measured using the ImageJ software. To analyze the silver grain density in von Kossa stained sections, micrographs at 400x magnification were imported into the ImageJ software package, and three 100 × 100 μm areas within the *Phospho1*^−/−^ and WT cuspal tip molar enamel region were randomly chosen and pixel density was analyzed. For EDS analysis, ten points were randomly chosen in the enamel region of three *Phospho1*^−/−^ and WT molars, and the values of calcium and phosphorus at the selected points was compared between both groups. All experiments were done in triplicates, the data analysis was conducted using student's *T*-test, and the significance value was set at *p* < 0.05.

## Results

### PHOSPHO1 protein localization in the enamel layer and the walls of secretory vesicles

**Figure 1:**
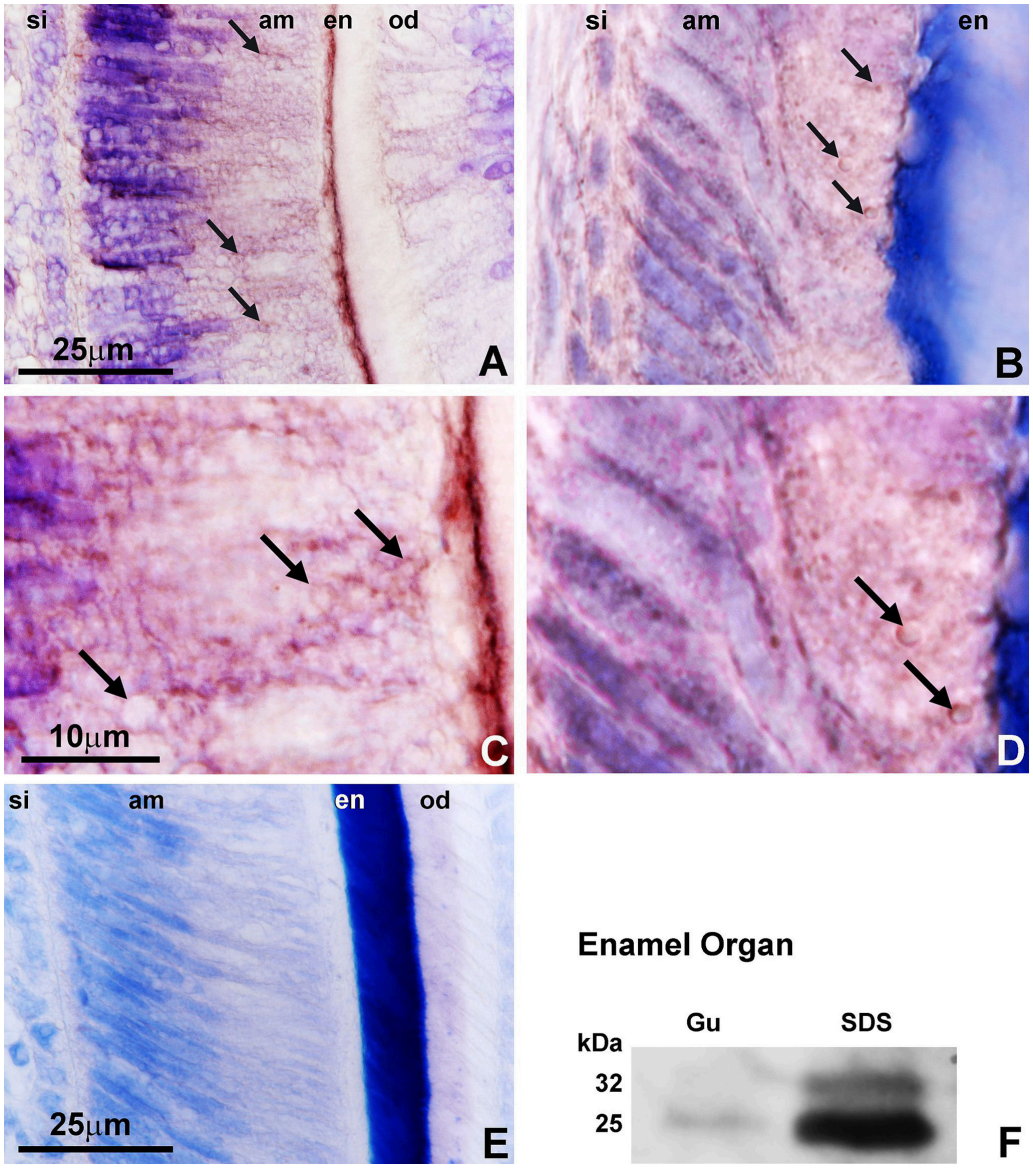
PHOSPHO1 localization in the ameloblast secretory vesicle walls. The first set of immunoreactions **(A,C)** was generated using a commercial antibody (GTX119275) and the second set was based on the original antibody from the Millán lab (AbD Serotec) **(B,D)**. Immunohistochemical labeling **(A–D)** located PHOSPHO1 at the periphery of secretory vesicles (arrows) and in the enamel layer of 3 days postnatal (dpn) WT mouse molars (en). **(C,D)** are higher magnification images of **(A,B)** allowing for detailed assessment of Phospho1 localization in ameloblast secretory vesicle walls (arrows). **(E)** is a same stage mouse molar control in which the primary antibody was replaced with pre-immune serum. **(F)** is a Western blot demonstrating the characteristic 32 KDa full-length PHOSPHO1 band and a 25 kDa cleavage product using the AbD Serotec antibody on blotted enamel organ extracts. Gu were 4M guanidine extracts and SDS were SDS extracts of enamel organ tissues. The scale bar was 25 μm for **(A,B,E)**, and 10 μm for **(C,D)**.

Immunohistochemical and Western blot analysis were performed to verify whether the PHOSPHO1 protein was localized in the enamel organ. Our analysis of paraffin sections of 3 days old wildtype mice revealed PHOSPHO1 protein localization in the enamel layer, Tomes' processes, and in ameloblast secretory vesicles (Figure 1). Positive signals for PHOSPHO1 were obtained using both antibodies available to us (GTX119275 from Genetex in Figures 1A,C and AbD Serotec/Millán in Figures 1B,D) and independent from the type of fixative employed (Formalin in Figures 1A,C,E, and Bouin in Figures 1B,D). In general, PHOSPHO1 expression levels were stronger in the mineralized enamel layer and at the ameloblast secretory pole (Figures 1A,C), while there was no staining in the control section using the same technique without primary antibody (Figure 1E). High magnification images revealed positive staining for PHOSPHO1 at the periphery of circular organelles at the ameloblast secretory pole (Figure 1C,D). A Western blot using the AbD Serotec antibody and performed on guanidine or SDS extracts of enamel organs demonstrated a characteristic 32 KDa full-length PHOSPHO1 band and a 25 kDa cleavage product, confirming the presence of PHOSPHO1 in the enamel organ.

### Decreased enamel mineralization and loss of sharp incisal tips in *Phospho1^−/−^* mice

**Figure 2:**
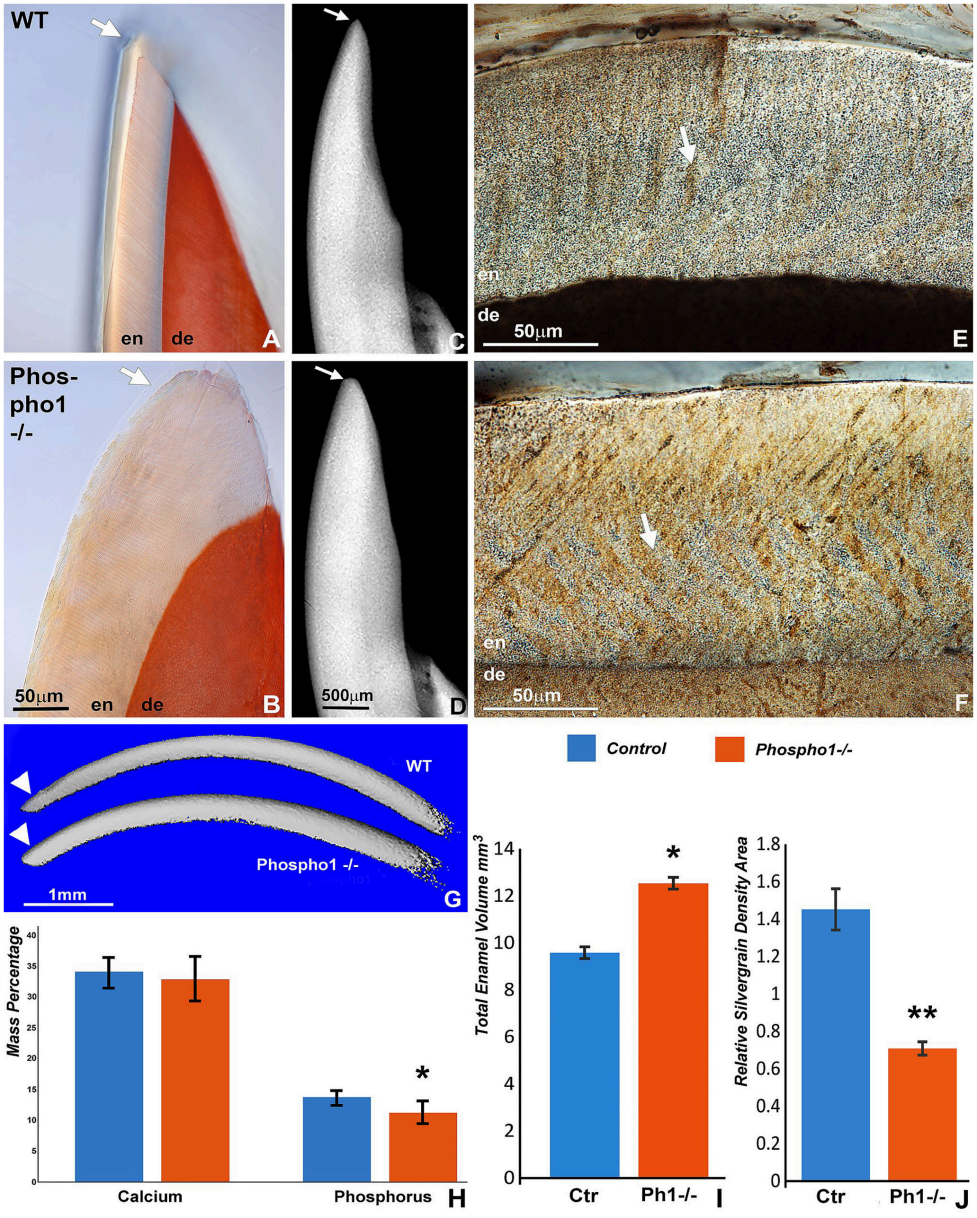
Decreased mineralization in the enamel layer of 30 days old *Phospho1*^−/−^ mice. Incisal tips of *Phospho1*^−/−^ mice **(B,D)** were thicker and rounded (arrows) when compared to those of WT mice. The molar enamel layer of *Phospho1*^−/−^ mice contained less von Kossa staining and more organic material in the prisms **(F)** than the WT mice **(E)**. Three-D micro-CT reconstructions of representative WT and *Phospho1*^−/−^ mouse incisors confirm differences in incisal tip contours and in the overall enamel volume **(G)**. Elemental analysis revealed a significant difference in the amount of phosphorus in the *Phospho1*^−/−^ mice when compared to the WT mice as demonstrated by EDS-SEM. The EDS-SEM data were calculated as average mass percent of calcium and phosphorus **(H)**. **(I)** Statistical analysis of the differences in enamel volume between WT and *Phospho1*^−/−^ mice based on 3D reconstructed micro-CT images. **(J)** Comparative analysis of differences in mineralization between WT and *Phospho1*^−/−^ mice as revealed by von Kossa stained ground sections using a grain counting algorithm. ^*^p < 0.05, ^**^p < 0.001. Control values are shown in blue columns and *Phospho1*^−/−^ data are displayed in orange/red colored columns. The scale bar is 50 μm for **(A,B)**, 500 μm for **(C,D)**, and 50 μm for **(E,F)**.

To determine how loss of PHOSPHO1 affects enamel quality, a number of physicochemical parameters such as total enamel volume, calcium, and phosphate ratios, level of mineralization based on von Kossa sections, and sharpness of incisal tips were assessed and compared between *Phospho1*^−/−^ and WT mice. Comparison of X-ray radiographs and alizarin red stained ground sections between 30 day old control and *Phospho1*^−/−^ mouse teeth revealed a rounded incisal tip in the PHOSPHO1 null incisors compared to the sharp incisal tips of their wild-type counterparts (Figures 2A–D). There was a significant 2-fold reduction in silver grain density on von Kossa stained ground sections (1.44 ± 0.11 vs. 0.71 ± 0.04 pixel units) indicative of reduced mineralization in the enamel layer of the *Phospho1*^−/−^ mice when compared to the WT mice (Figures 2E,F,J). Von Kossa staining also demonstrated a substantial reduction in dentin mineralization in the *Phospho1*^−/−^ mice vs. controls (Figures 2E,F). Total enamel volume as measured by micro-CT volumetric analysis was significantly higher in *Phospho1*^−/−^ incisors compared to WT controls (Figures 2G,I). EDS elemental analysis demonstrated a significant decrease in the amount of phosphorus in the *Phospho1*^−/−^ mice when compared to controls (*p* < 0.05), while calcium levels were only little affected by the loss of PHOSPHO1 (Figure 2H).

### Disintegration and loss of enamel prism structure in *Phospho1^−/−^* mouse enamel

**Figure 3:**
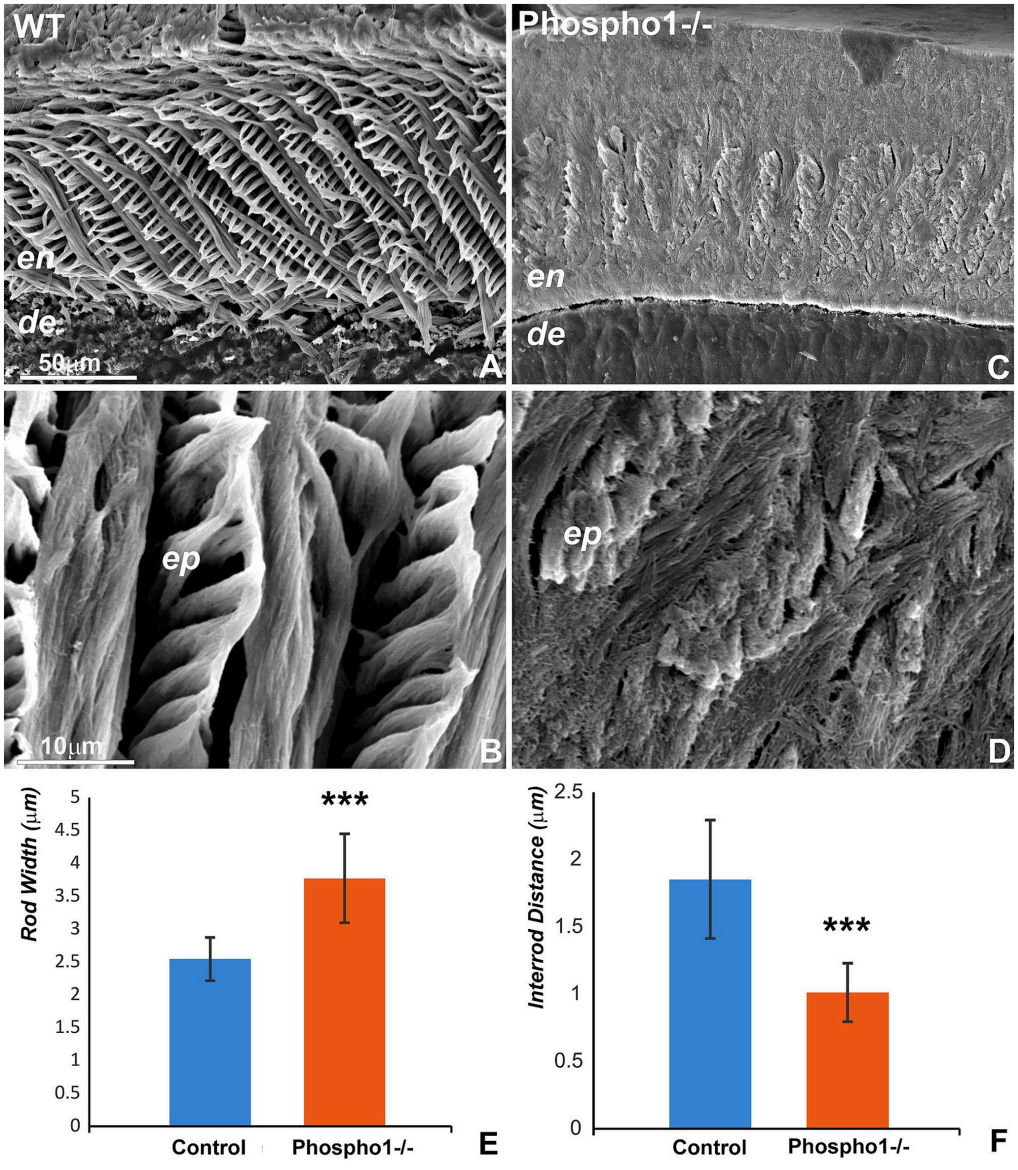
Loss of enamel prism structure and crystal organization in *Phospho1*^−/−^ mouse molars. Scanning electron micrographs of 30 days old mouse molars illustrate the arrangement of enamel prims at 750x and 5000x magnifications (**A,B**, respectively) in WT **(A,B)** and *Phospho1*^−/−^ **(C,D)** mouse molars. There was a significant loss of enamel prism structure as well as a dramatic change in the organization of the enamel crystals in the PHOSPHO1 null mice. **(E,F)** Morphometric comparison of enamel prism width **(E)** and inter-rod distance **(F)** between WT and *Phospho1*^−/−^ mice. ^***^p < 0.0001. The scale bar is 50 μm for **(A,C)** and 10 μm for **(B,D)**.

Scanning electron microscopy comparisons between 30 days old *Phospho1*^−/−^ and control mice demonstrated a complete loss of the prominent enamel prism “picket fence” structure on surface etched *Phospho1*^−/−^ mouse enamel preparations when compared to controls (Figures 3A–D). High magnification scanning electron micrographs at individual crystal resolution revealed a loss of individual prism boundaries as a result of a 45.3% reduction in the mineral-free inter-prism space between individual prisms (Figure 3F) in *Phospho1*^−/−^ mice when compared to controls (Figures 3C,D vs. Figures 3A,B). These micrographs also documented a loss of parallel crystal organization within prisms (Figure 3D). Moreover, there was a 1.56-fold increase in enamel prism width (Figure 3E) in *Phospho1*^−/−^ mutant mice (*p* < 0.0001).

### Presence of mineral precipitates in ameloblast secretory vesicles as revealed by transmission electron microscopy

**Figure 4:**
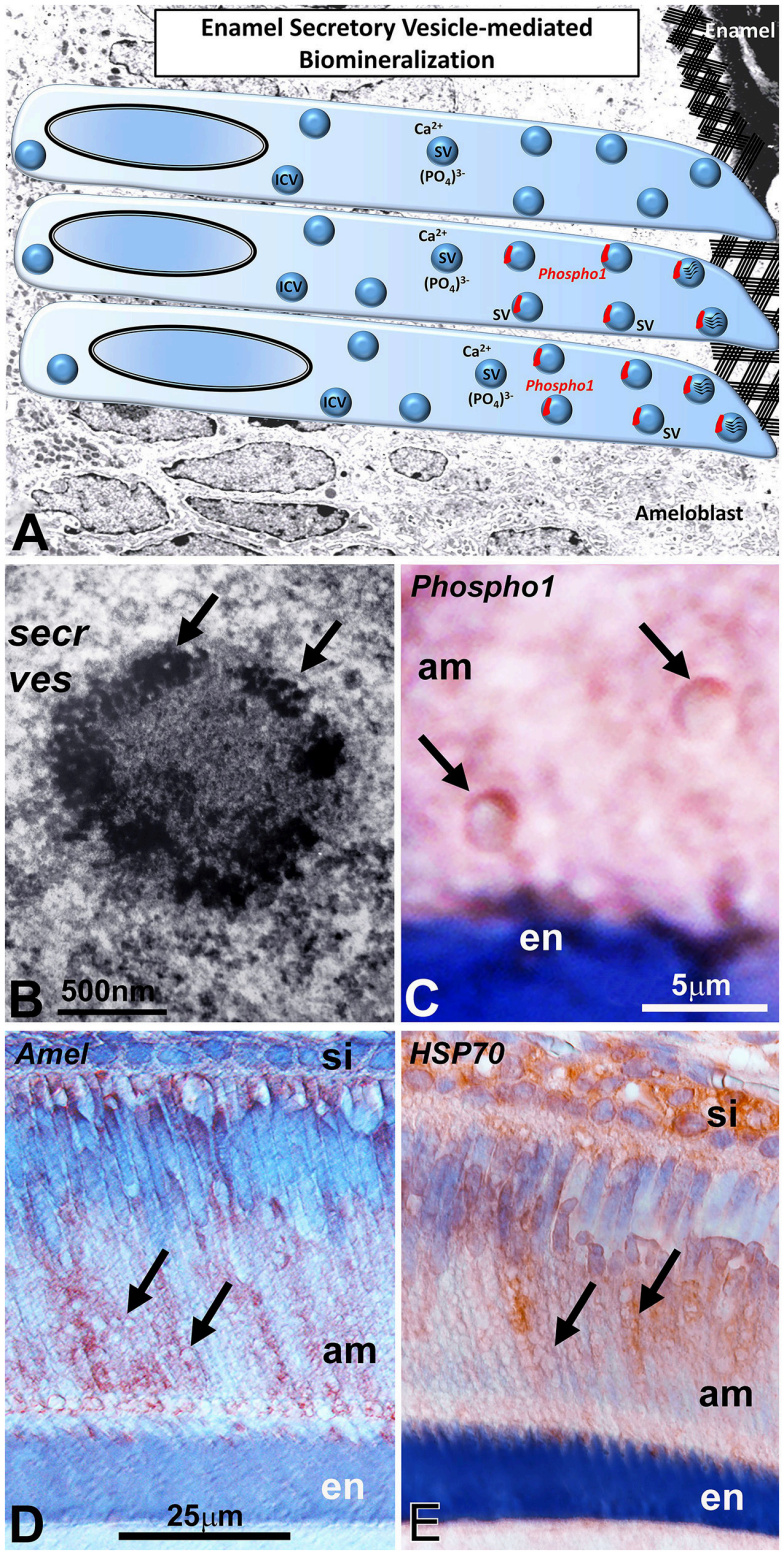
PHOSPHO1 as an intravesicular apatite nucleation enzyme in ameloblast secretory vesicles **(A)**. The presence of PHOSPHO1 as a secretory vesicle (SV) component in ameloblast secretory vesicles suggests intravesicular mineral/matrix assembly prior to secretion into the ameloblast extracellular matrix. The transmission electron micrograph **(B)** of ameloblast intracellular secretory vesicles supports the concept of mineral nucleation sites (arrows) within such secretory vesicles (secr ves). This secretory vesicle was localized at the secretory face of the ameloblast. **(C)** is a high magnification micrograph demonstrating highly specific immunostaining for PHOSPHO1 in the walls of ameloblast secretory vesicles (arrows). **(D,E)** are immunoreactions using either anti-amelogenin **(D)** or anti-HSP70 antibodies **(E)**, demonstrating immunoreactivity associated with secretory vesicles (arrows). secr ves, secretory vesicles; am, ameloblasts; si, stratum intermedium; en, enamel. The scale bar is 500 nm **(B)**, 5 μm for **(A,C)** and 25 μm for **(D,E)**.

Ameloblast secretory vesicles (sketch in Figure 4A) were long considered to be protein-rich intracellular entities within ameloblasts. Micrographs presented in this study illustrate that following 12 days of organ culture, these secretory vesicles also contained numerous mineral precipitates at the outside of and within the secretory vesicles (Figure 4B). Moreover, high magnification immunohistochemistry micrographs demonstrated the presence of half-moon shaped PHOSPHO1-positive signals in the walls of ameloblast secretory vesicles close to the developing enamel front (Figure 4C). Supportive of their involvement in secretory processes during amelogenesis, vesicles also stained positively for amelogenin and the vesicular marker protein HSP70 on paraffin sections of secretory ameloblasts (Figures 4D,E).

## Discussion

The purpose of the present study was to determine the function of the matrix vesicle phosphatase PHOSPHO1 during amelogenesis and on the structural integrity of the enamel layer. In this study we compared the enamel layers of wild-type and *Phospho1*^−/−^ mice using light and electron microscopy as well as elemental mapping and von Kossa staining. Three different models have been used to verify PHOSPHO1 expression and function during amelogenesis: (i) the *Phospho1*^−/−^ mouse molar model for phenotype analysis and immunohistochemistry, (ii) E16 mouse molar organ culture to visualize secretory vesicles using electron microscopy, (iii) and porcine enamel organ protein extracts to verify the presence of PHOSPHO1 in the enamel organ. In general, our study demonstrated reduced enamel mineralization and prism formation as a results of loss of PHOSPHO1. Using immunohistochemistry, PHOSPHO1 was localized to the walls of ameloblast secretory vesicles and in the enamel layer. Together, these findings demonstrate that PHOSPHO1 is essential for physiological enamel ion transport and for the structure of the enamel layer.

Our immunochemical analysis using two different antibodies against PHOSPHO1 and two different types of fixation procedures demonstrated that PHOSPHO1 was localized in the enamel layer and adjacent to the walls of ameloblast secretory vesicles. The presence of PHOSPHO1 in the walls of ameloblast secretory vesicles was a surprise finding as ameloblast vesicles are commonly distinguished from the matrix vesicles involved in the mineralization of bone, dentin, and cartilage (Nanci, 2008). The presence of PHOSPHO1 in ameloblast vesicles would suggest potential similarities between matrix vesicles and ameloblast secretory vesicles as PHOSPHO1 functions to facilitate initial mineralization inside of such vesicles. PHOSPHO1 presence in ameloblast vesicles was previously documented by McKee et al. (2013), and the substantial enamel defects in mice lacking the PHOSPHO1 enzyme reported here support the concept of a putative function of PHOSPHO1 during amelogenesis. Similar to matrix vesicles, ameloblast secretory vesicles are surrounded by a distinct membrane and contain a mixture of protein matrix and small mineral crystallites (Diekwisch, 1998; Brookes et al., 2014), even though they lack the double layer membrane commonly associated with matrix vesicles (Hsu and Anderson, 1978). There was also an obvious size difference between the ameloblast secretory vesicles described here (2–3 μm) and the average diameter of typical matrix vesicles of the bone extracellular matrix (100 nm, Anderson, 2003), and this size difference may be explained by the function of ameloblasts to transport and deposit substantial amounts of protein and mineral within a relatively short period of time. Nevertheless, further studies will be needed to establish the similarities and differences between the matrix vesicles of the mesenchymal bone extracellular matrix and the secretory vesicles of ameloblast epithelial cells beyond the differences in localization, content, and size. Especially, cryoimmuno-electron microscopic techniques to map individual matrix components within individual vesicle components would further advance our understanding of the secretory machinery common to epithelial and mesenchymal mineral forming cells. While the presence of PHOSPHO1 in ameloblast secretory vesicles alone may not suffice to draw parallels between matrix vesicles and ameloblast vesicles, PHOSPHO1 as a common apatite nucleation enzyme in both types of vesicles suggests similar mechanisms of mineral growth related to early intravesicular apatite nucleation between mineralized tissues of ectodermal and ectomesenchymal origin (Figure 4A).

Loss of *Phospho1* in our mouse model had a number of dramatic effects on enamel prism structure and mineralization, including reduced mineralization as indicated by von Kossa staining, rounded incisal tips, disrupted enamel prism pattern, and lack of individual crystal integrity within their prismatic subunit. Together, this phenotype indicates that enamel biomineralization in *Phospho1*^−/−^ mice was severely impaired. Based on the present phenotype it is not entirely clear whether the dramatic phenotype in the mature enamel was due to a failure of mineralization initiation or due to a failure of PHOSPHO1 to continuously function during enamel maturation within the enamel layer. It is also not clear whether PHOSPHO1 plays only a direct role in early crystal nucleation and growth, and the lack of sufficiently hardened crystals impairs their assembly into enamel prisms, or whether PHOSPHO1 has additional functions during prism organization. Nevertheless, the remarkable enamel phenotype reported in the present study confirms that a phosphatase so far only known for its role during the early phase of bone and dentin crystal growth is essential for physiological enamel mineralization and prism patterning. Moreover, our electron micrographs of intravesicular mineral precipitation within ameloblast secretory vesicles support the concept of PHOSPHO1 function during initial mineralization.

In addition to the mineralization defects discussed above, there was also a significant increase in overall enamel volume and in the thickness of the enamel layer. This is a remarkable finding as the thickness of the enamel layer is usually highly consistent within a species. Yet, the reduced level of mineralization might have caused a delayed onset of enzymatic degradation of the enamel matrix via enamel-related proteases such as MMP20 and KLK4. Alternatively, the higher level of mineralization in the wild-type mice might have been associated with a greater degree of protein compaction and a resulting thinner enamel layer. The less mineralized enamel layer also explains the rounded incisal tips of *Phospho1*^−/−^ mice, where a different mineral content results in an altered wear pattern. Finally, the reduced level of underlying dentin mineralization might have affected the mechanosensory control of the ameloblast secretory apparatus, resulting in an overcompensation through enhanced enamel secretion.

While our data revealed remarkable structural defects in the enamel layer of *Phospho1*^−/−^ mice, our elemental analysis demonstrated that only phosphate incorporation into the enamel layer of *Phospho1*^−/−^ mice was significantly reduced, and calcium levels were not significantly different between mutant and wild-type mice. Phosphate content is essential for normal bone health, and loss of phosphate is destined to set the physiological calcium/phosphate homeostasis out of balance (Penido and Alon, 2012). However, the role of PHOSPHO1 is predominantly associated with early intravesicular mineral nucleation, suggesting the phenotype observed here is less due to a reduction in phosphate transport but rather because of the failure of early intravesicular crystals to form as precursors and templates for mature enamel crystals and subsequently enamel prisms.

## Ethics statement

All animal studies were approved by and conducted in accordance with the guidelines of the Texas A&M University College of Dentistry Animal Care Committee.

## Author contributions

MP and TD wrote the manuscript, TD, JM, and CF designed the experiments, and MP and LR conducted the experiments.

### Conflict of interest statement

The authors declare that the research was conducted in the absence of any commercial or financial relationships that could be construed as a potential conflict of interest.
